# Supplementary material for: Did Covid-19 lockdown positively affect the urban environment and UN- Sustainable Development Goals?
Source: PLoS One. 2022 Sep 23;17(9):e0274621. doi: 10.1371/journal.pone.0274621 (PMC9506620; doi:10.1371/journal.pone.0274621)
Supplement: S1 File — (DOCX) [file pone.0274621.s001.docx]

**Did Covid-19 lockdown positively affect the urban environment and UN- Sustainable Development Goals?**

Ritwik Nigam^1^, Gaurav Tripathi^2^, Tannu Priya^2^, Alvarinho J. Luis^3^, Eric Vaz^4^, Shashikant Kumar^5^, Achala Shakya^6^, Bruno Damásio^7*^, Mahender Kotha^1^

^1^School of Earth, Ocean and Atmospheric Sciences (SEOAS), Goa University, Goa, India, 403004

^2^Department of Geoinformatics, Central University of Jharkhand, Ranchi, Jharkhand, India, 835205

^3^Polar Remote Sensing Section, National Centre of Polar and Ocean Research, Ministry of Earth Science, Govt. of India, Headland Sada, Goa- 403804, India.

^4^Department of Geography and Environmental Studies, Ryerson University, Toronto, Ontario, M5B 2K3, Canada

^5^Department of Architecture, Parul University, Limda, Gujarat, India, 391760

^6^Department of Computer Engineering, National Institute of Technology, Kurukshetra, Haryana, India, 136119

^7^NOVA Information Management School (NOVA IMS), Universidade Nova de Lisboa, Campus de Campolide, 1070-312 Lisboa, Portugal

^*^Corresponding author: Bruno Damásio (email: bdamasio@novaims.unl.pt)

**Supplementary material**

**CReDiT authorship contribution statement:**

**Ritwik Nigam:** Conceptualization, Methodology, Discussion, Formal analysis, original draft preparation; **Gaurav Tripathi:** Methodology, Data processing, Data visualization; **Tannu Priya**: Data Processing, Data Visualization; **Alvarinho Luis**: Formal analysis, Interpretation, Review, and Editing; **Eric Vaz:** Data analysis, Review and Editing; **Achala Shakya:** Data processing; **Shashikant Kumar:** Data analysis; **Corresponding author Bruno Damasio:** Data analysis, Review, and Editing; **Mahender Kotha:** Formal analysis, Visualization, Interpretation, Review, and Editing; corresponding author Bruno: Review and Editing

## **Declaration of competing interest:**

The authors report no potential conflict of interest and declare that they have no known competing financial interests or personal relationships that could have appeared to influence the work reported in this paper.

## **Data availability**

## The data supporting the findings of this study are available in the Supplementary Information. Additional information can be obtained from collected from the corresponding author.

Supplementary Table 3. Characteristics of NDVI, NDWI, LST and NTL variables for 2020 period with respect to similar period of 2019.

| **City** | **Parameters** | **Period** | **Pixel Difference** | **%Pixel difference** | **Peak SNR** | **SSIM** | **Correlation Coefficient** | **%Mean difference** |
| --- | --- | --- | --- | --- | --- | --- | --- | --- |
| **Kuala Lumpur** | **NDVI** | Pre | 0.12 | 69.44 | 14.21 | 0.71 | 0.82 | 16% |
|  |  | During | 0.12 | 68.52 | 13.60 | 0.62 | 0.72 | 7700% |
|  |  | Post | 0.13 | 67.10 | 12.80 | 0.59 | 0.68 | -4117% |
|  | **NDWI** | Pre | 0.01 | 3.30 | 22.30 | 0.86 | 0.78 | 2777% |
|  |  | During | 0.00 | 0.00 | Inf | 1.00 | 1.00 | 0% |
|  |  | Post | 0.02 | 3.34 | 18.85 | 0.82 | 0.52 | 1428% |
|  | **LST** | Pre | 0.00 | 0.00 | Inf | 1.00 | 1.00 | -1264% |
|  |  | During | 0.00 | 0.00 | Inf | 1.00 | 1.00 | 42% |
|  |  | Post | 0.00 | 0.00 | Inf | 1.00 | 1.00 | 272% |
|  | **NTL** | Pre | 0.00 | 0.00 | Inf | 1.00 | 1.00 | -960% |
|  |  | During | 0.00 | 0.00 | Inf | 1.00 | 1.00 | -964% |
|  |  | Post | 0.17 | -17.12 | 7.66 | 0.80 | 0.69 | -2066% |
| **Mexico** | **NDVI** | Pre | 0.05 | 55.89 | 19.96 | 0.82 | 0.95 | 17% |
|  |  | During | 0.05 | 56.10 | 19.21 | 0.86 | 0.93 | -2272% |
|  |  | Post | 0.06 | 56.27 | 17.59 | 0.82 | 0.94 | 1020% |
|  | **NDWI** | Pre | 3.42 | 0.94 | 45.32 | 0.99 | 0.69 | 384% |
|  |  | During | 1.11 | 0.25 | 48.85 | 1.00 | 0.57 | 357% |
|  |  | Post | 2.21 | 0.36 | 45.44 | 1.00 | 0.47 | 1470% |
|  | **LST** | Pre | 0.00 | 0.00 | Inf | 1.00 | 1.00 | 1199% |
|  |  | During | 0.00 | 0.00 | Inf | 1.00 | 1.00 | 1685% |
|  |  | Post | 0.00 | 0.00 | Inf | 1.00 | 1.00 | 109% |
|  | **NTL** | Pre | 0.01 | -16.14 | 27.52 | 0.96 | 1.00 | 119% |
|  |  | During | 0.03 | -15.25 | 20.41 | 0.95 | 0.98 | 624% |
|  |  | Post | 0.02 | -18.64 | 25.88 | 0.95 | 0.99 | 781% |
| **Greater Mumbai** | **NDVI** | Pre | 0.04 | 47.97 | 22.95 | 0.91 | 0.97 | -41% |
|  |  | During | 0.04 | 48.09 | 23.02 | 0.92 | 0.97 | 0% |
|  |  | Post | 0.09 | 48.87 | 14.17 | 0.76 | 0.81 | 2187% |
|  | **NDWI** | Pre | 0.01 | 2.81 | 25.91 | 0.94 | 0.92 | 3125% |
|  |  | During | 0.01 | 2.66 | 22.06 | 0.93 | 0.79 | 4166% |
|  |  | Post | 0.01 | 3.81 | 29.23 | 0.93 | 0.97 | 0% |
|  | **LST** | Pre | 0.01 | -1.00 | 20.02 | 0.93 | 0.98 | -2244% |
|  |  | During | 0.01 | -0.74 | 21.31 | 0.95 | 0.98 | -286% |
|  |  | Post | 0.00 | -0.35 | 24.52 | 0.98 | 0.99 | 26% |
|  | **NTL** | Pre | 0.00 | 0.00 | Inf | 1.00 | 1.00 | -1186% |
|  |  | During | 0.00 | 0.00 | Inf | 1.00 | 1.00 | -1055% |
|  |  | Post | 0.00 | -0.59 | 24.86 | 0.99 | 0.99 | 5191% |
| **Sao Paulo** | **NDVI** | Pre | 0.10 | 44.16 | 13.09 | 0.66 | 0.73 | -21% |
|  |  | During | 0.06 | 44.46 | 16.54 | 0.80 | 0.88 | 967% |
|  |  | Post | 0.02 | 42.60 | 25.99 | 0.95 | 0.99 | 571% |
|  | **NDWI** | Pre | 0.00 | 1.38 | **26.58** | 0.96 | 0.92 | -312% |
|  |  | During | 0.00 | 2.44 | **26.39** | 0.96 | 0.92 | 357% |
|  |  | Post | 0.00 | 2.39 | **30.91** | 0.98 | 0.97 | -357% |
|  | **LST** | Pre | 0.00 | 0.00 | Inf | 1.00 | 1.00 | -23% |
|  |  | During | 0.00 | 0.00 | Inf | 1.00 | 1.00 | -167% |
|  |  | Post | 0.00 | 0.00 | Inf | 1.00 | 1.00 | -250% |
|  | **NTL** | Pre | 0.01 | 4.54 | 30.01 | 0.98 | 1.00 | -333% |
|  |  | During | 0.01 | 4.68 | 30.50 | 0.99 | 1.00 | 1984% |
|  |  | Post | 0.01 | 5.17 | 28.24 | 0.98 | 1.00 | -4455% |
| **Toronto** | **NDVI** | Pre | 0.13 | 48.43 | 12.63 | 0.51 | 0.39 | -500% |
|  |  | During | 0.09 | 47.78 | 14.71 | 0.64 | 0.64 | -1818% |
|  |  | Post | 0.14 | 48.80 | 11.85 | 0.61 | 0.58 | -15454% |
|  | **NDWI** | Pre | 0.01 | 5.62 | 25.72 | 0.90 | 0.91 | -1666% |
|  |  | During | 0.00 | 1.99 | 31.93 | 0.98 | 0.98 | -740% |
|  |  | Post | 2.70 | 1.59 | 30.63 | 0.98 | 0.97 | 370% |
|  | **LST** | Pre | 0.29 | -29.49 | 5.30 | 0.44 | 0.79 | 29872% |
|  |  | During | 2.00 | -18.23 | 15.22 | 0.85 | 0.81 | -1690% |
|  |  | Post | 3.89 | -0.04 | 34.11 | 1.00 | 1.00 | -1173% |
|  | **NTL** | Pre | 0.00 | 0.00 | Inf | 1.00 | 1.00 | -5773% |
|  |  | During | 0.00 | 0.00 | Inf | 1.00 | 1.00 | -697% |
|  |  | Post | 0.00 | 0.00 | Inf | 1.00 | 1.00 | 914% |

Supplementary figures


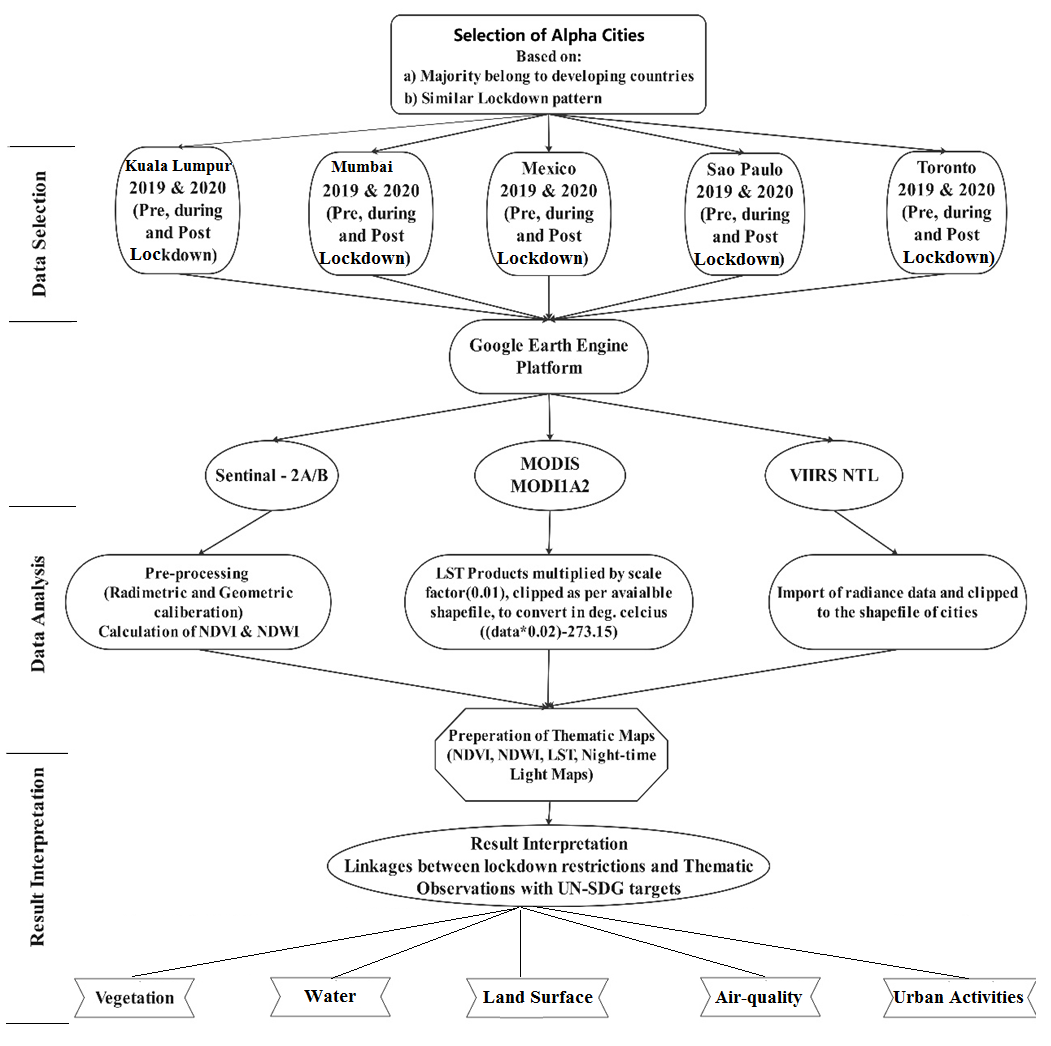


Supplementary Figure 1. Workflow adopted for processing satellite data in the present study.


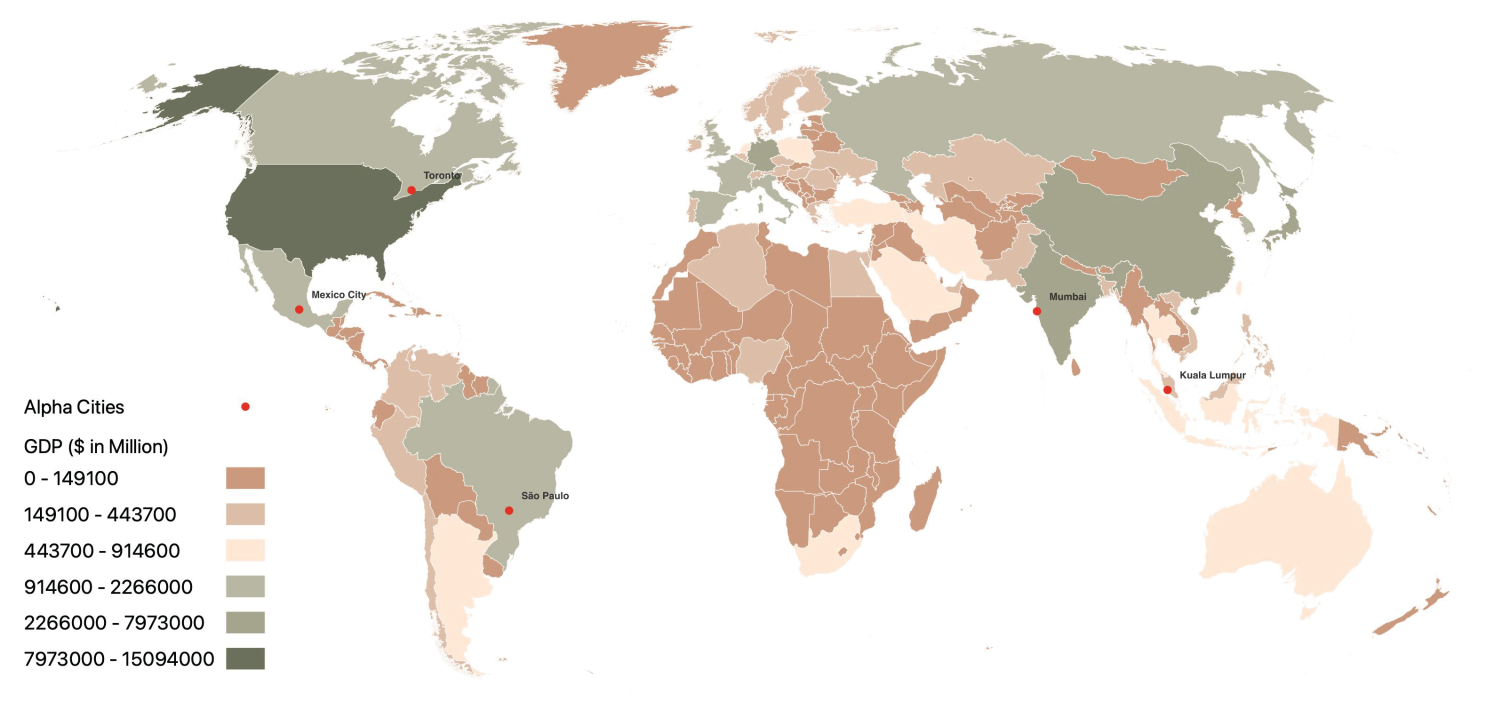


Supplementary Figure 2. Location of the Alpha Cities.

Supplementary tables

| Supplementary Table-1. List of data used for each environmental variables along with data acquisition dates | | | | | | |
| --- | --- | --- | --- | --- | --- | --- |
| **Dataset** | **Spatial**  **Resolution** | **City** | **Lockdown phase** | **Acquisition date** | **Source** | **Purpose** |
| **MODIS MOD11A2**  LST DAILY L3 GLOBAL DATA | 1 km | KUALA LUMPUR | Pre | 5^th^ & 7^th^ March 2019 & 2020 | GEE | **LST mapping** |
|  |  |  | During | 12^th^ and 15^th^ April 2019 & 2020 |  |  |
|  |  |  | Post | June 28 & July 01 2019 & 2020 |  |  |
|  |  | MEXICO | Pre | 9^th^ & 14^th^ March 2019 &2020 |  |  |
|  |  |  | During | 22^nd^ & 16^th^ April 2019 & 2020 |  |  |
|  |  |  | Post | 14^th^ & 20^th^ August 2019 & 2020 |  |  |
|  |  | GREATER MUMBAI | Pre | 9^th^ & 11^th^ March 2019 & 2020 |  |  |
|  |  |  | During | 12^th^ & 13^th^ April 2019 & 2020 |  |  |
|  |  |  | Post | 16^th^ & 16^th^ August 2019 & 2020 |  |  |
|  |  | SAO PAULO | Pre | 10^th^ & 11^th^ March 2019 & 2020 |  |  |
|  |  |  | During | 11^th^ & 13^th^ April 2019 & 2020 |  |  |
|  |  |  | Post | 8^th^ & 11^th^ July 2019 & 2020 |  |  |
|  |  | TORONTO | Pre | 7^th^ & 8^th^ March 2019 & 2020 |  |  |
|  |  |  | During | 16^th^ & 21 April 2019 & 2020 |  |  |
|  |  |  | Post | 8^th^ & 10^th^ July 2019 & 2020 |  |  |
| **SENTINEL-2A** | 10 m | KUALA LUMPUR | Pre | 8^th^ & 9^th^ March 2019 & 2020 | GEE | **NDVI and NDWI mapping** |
|  |  |  | During | 13^th^ & 14^th^ April 2019 & 2020 |  |  |
|  |  |  | Post | June 28 & July 29 2019 & 2020 |  |  |
|  |  | MEXICO | Pre | 12^th^ & 13^th^ March 2019 & 2020 |  |  |
|  |  |  | During | 14^th^ & 25^th^ April 2019 & 2020 |  |  |
|  |  |  | Post | 18^th^ & 19^th^ August 2019 & 2020 |  |  |
|  |  | MUMBAI | Pre | 9^th^ & 11^th^ March 2019 & 2020 |  |  |
|  |  |  | During | 12^th^ & 11^th^ April 2019 & 2020 |  |  |
|  |  |  | Post | 16^th^ & 10^th^ August 2019 & 2020 |  |  |
|  |  | SAO PAULO | Pre | March 11 2019 & 2020 |  |  |
|  |  |  | During | April 12 2019 & 2020 |  |  |
|  |  |  | Post | 10^th^ & 11^th^ July 2019 & 2020 |  |  |
|  |  | TORONTO | Pre | 6^th^ & 7^th^ March 2019 & 2020 |  |  |
|  |  |  | During | 22^nd^ & 23^rd^ April 2019 & 2020 |  |  |
|  |  |  | Post | 9^th^ & 10^th^ July 2019 & 2020 |  |  |
| **VIIRS NTL** | 15 arc second | KUALA LUMPUR | Pre | 3^rd^ & 6^th^ March 2019 & 2020 | GEE | **Night Time Light data** |
|  |  |  | During | 14^th^ & 20^th^ April 2019 & 2020 |  |  |
|  |  |  | Post | June ^26 &^ July 04 2019 & 2020 |  |  |
|  |  | MEXICO | Pre | 6^th^ & 11 March 2019 & 2020 |  |  |
|  |  |  | During | 23^rd^ & 12^th^ April 2019 & 2020 |  |  |
|  |  |  | Post | 18^th^ & 13^th^ August 2019 & 2020 |  |  |
|  |  | MUMBAI | Pre | 9^th^ & 10^th^ March 2019 & 2020 |  |  |
|  |  |  | During | 11^th^ & 12^th^ April 2019 & 2020 |  |  |
|  |  |  | Post | 14^th^ & 10^th^ August 2019 & 2020 |  |  |
|  |  | SAO PAULO | Pre | 12^th^ & 11^th^ March 2019 & 2020 |  |  |
|  |  |  | During | 12^th^ & 11^th^ April 2019 & 2020 |  |  |
|  |  |  | Post | 9^th^ & 10^th^ July 2019 & 2020 |  |  |
|  |  | TORONTO | Pre | 5^th^ & 10^th^ March 2019 & 2020 |  |  |
|  |  |  | During | 19^th^ & 17^th^ April 2019 & 2020 |  |  |
|  |  |  | Post | 9^th^ & 10^th^ July 2019 & 2020 |  |  |

Supplementary Table 2. General characteristics of alpha cities.

|  | **Kuala-Lumpur**  (03˚08’52”N; 101˚41’43”E) | **Greater Mumbai**  (18˚58’30” N; 72˚49’33” E) | **Mexico City**  (19˚26' N;  99˚8' W) | **Sao Paulo**  (23˚33’S; 46˚38’W) | **Toronto**  (43˚44’30” N; 79˚22'24" W) |
| --- | --- | --- | --- | --- | --- |
| **General** | Malaysia's capital and largest city with an area of 243 sq. km. It is a cultural, financial and economic centre^32^. Population is 1.76 million. | Financial capital of India, 7^th^ most populous city in the world^34^ with around 20 million population and area of 603 sq. km | Capital of Mexico and the largest city on the North American continent^37^ with an area of 1485 sq. km and a population of 8.92 million. | A megacity in Brazil, most populous city in the whole of Western and Southern hemispheres^41^ with an area of 1521 sq. km; it is the 4^th^ largest city of the world in terms of population (12.17 million). | Capital of Ontario province and the largest city in Canada, covering an area of 630 sq. km.^44^ |
| **Climate** | A tropical rainforest climate, with temperatures ranging between 35° and 23.4°C. It receives a minimum of 10 cm of rainfall annually, with the least amount during May to August | A tropical wet and dry climate with heavy monsoon (242.2 cm annually) from June to Sept; it is located on a narrow peninsula in the Arabian Sea, its suburbs located on mainland | A tropical highland climate, with the mean temperature varies between 12˚ to 16˚C^38,39^. | A humid subtropical climate with a mean annual temperature between 19˚ to 28˚C ^42^. | A hot-summer humid continental climate^45^, temperature ranges between ‒7˚C to 27˚C annually. |
| **Economic** | Ranked second after Singapore in Southeast Asia for trade connectivity and 70^th^ globally by the Economist Intelligence Units' Global Livability Ranking^33^. | Placed in the top-10 list of the important commercial centers of the world^35^ in terms of global financial inflow. City is known for air pollution which is worse than in Delhi^36^. | One of the prominent socio-cultural and financial cities of the world^40^ and one of highest polluted cities in North America, with ozone pollution 2.5 times higher than the WHO's limits15. | It has11^th^ largest Gross Development Product (GDP) in the world^43^. | It is important financial center of Canada and is regarded as one of the most multicultural and cosmopolitan cities in the world^46^. |
